# Impaired wound healing in Parkinson’s disease: a hypothesis on altered epidermal growth factor (EGF) and N-methyl-D-aspartate (NMDA) signaling in keratinocytes

**DOI:** 10.1186/s10020-025-01247-w

**Published:** 2025-05-22

**Authors:** Caroline Liu, Johanna Ghebrehiwet-Kuflom, Roslyn Rivkah Isseroff, Sara Dahle, Vera Morhenn

**Affiliations:** 1 University of California Davis School of Medicine, 4610 X St, Sacramento, CA 95817 USA; 2 Dermatology Service, VA Northern California, Sacramento VA Medical Center, 10535 Hospital Way, Mather, CA 95655-4200 USA; 3 Department of Dermatology, University of California Davis Medical Center, 4301 X St, Sacramento, CA 95817 USA; 4 Podiatry Service, VA Northern California, Sacramento VA Medical Center, 10535 Hospital Way, Mather, CA 95655-4200 USA

**Keywords:** Wound healing, Parkinson’s disease, NMDA, EGF, Psoriasis

## Abstract

Parkinson’s Disease (PD) is a neurodegenerative disorder characterized by the depletion of dopaminergic neurons in the substantia nigra, leading to hallmark motor symptoms such as bradykinesia, tremor, and rigidity. While the focus of PD has been on motor changes, dermatological changes are also commonly seen and may even precede the neurological symptoms. Individuals with PD may exhibit impaired wound healing, potentially due to dysregulated mechanisms involving epidermal growth factor (EGF) and N-methyl-D-aspartate (NMDA) in keratinocytes. This paper hypothesizes that the potential for impaired wound healing in PD patients is linked to reduced EGFR activity and altered NMDAR subunit expression in keratinocytes, in contrast to the upregulated wound healing seen in conditions like psoriasis, which demonstrates elevated EGFR and changes in NMDAR subunit activity. Furthermore, a potential co-interaction between EGF and NMDA in keratinocytes may further contribute to impaired wound healing. Investigating these signaling mechanisms can improve understanding and management of associated dermatological symptoms. We propose additional studies to quantify differences in rates of wound healing between PD patients and age-matched controls in effort to explore therapeutic targets for enhancing wounding healing in the context of PD.

## Introduction

Parkinson’s disease (PD) is a progressive neurodegenerative disorder of the substantia nigra, depleting dopaminergic neurons, leading to its hallmark effects of motor dysfunction. Common motor presentations include bradykinesia, tremor, postural instability, and rigidity. Some non-motor symptoms include cognitive decline, depression and anxiety, incontinence, constipation, and a variety of skin disorders. The dermatological changes associated with PD are less studied despite being commonly seen across affected patients and may even precede the neurological symptoms (Ravn et al. 2017). Some of these changes include seborrheic dermatitis, dry skin, and melanoma (Beitz 2013). Current literature documents increased postoperative surgical site, superficial, and periprosthetic wound infection in the context of total joint arthroplasty that may impair wound healing in PD patients (Wang et al. 2023). However, the healing process itself and the reasons why have not yet been examined. Additionally, skin manifestations within neurological conditions are not limited to PD. Multiple sclerosis (MS) has been associated with distinct skin presentations, which may offer insights into the intersection of the nervous system and skin. MS patients can develop sclerosing skin disorders, such as systemic sclerosis, and exhibit increased sodium content in the skin, which has been linked to disease activity (Quattrocchi et al. 2023). Autoimmune skin disorders such as pemphigus and pemphigoid, which cause blistering of the skin and mucous membranes, have been reported with greater frequency in patients with Alzheimer’s disease (Xie et al. 2023). Given that several neurological conditions demonstrate skin manifestations that are not mechanistically well-studied, we propose a hypothesis to explore how molecular and cellular differences in the skin of PD patients, compared to unaffected individuals, may underlie or contribute to impaired healing.

The impact of PD on wound healing may stem from several factors. There may be a greater risk of falls and physical trauma associated with postural instability and gait changes. Pressure, friction, and moisture from difficulty moving or residing in bed long-term affects microcirculation and increases the risk for ischemic skin damage and pressure ulcers. Urinary and fecal incontinence can lead to moisture-associated skin damage and incontinence-associated dermatitis (Beitz 2013). Patients may also have a more difficult time tending to wounds due to rigidity in movement and bradykinesia (Saikia et al. 2020). Medication side effects can further contribute to skin breakdown. Processes such as inflammation, angiogenesis, and tissue oxygenation may be dysregulated due to autonomic dysfunction. We focus on the molecular and cellular differences in the skin of unaffected individuals compared to PD patients, as noted in detail below, for a proposed hypothesis of impairment of wound healing. However, we must keep in mind the influences of the abovementioned confounding factors to wound healing in PD.

While the focus of PD research has been on the pathophysiology in neurons, characterized by an increase in N-methyl-d-aspartate (NMDA) activity in the brain, there have been noted changes to NMDA signaling in cultured epidermal keratinocytes, suggesting that similar changes might be present in PD epidermis (Nahm et al. 2004). In addition, epidermal growth factor (EGF) signaling is crucial to the process of wound healing. While psoriasis has been studied as a skin condition characterized by increased rates of wound healing due to upregulation of epidermal growth factor receptor (EGFR) and certain subunits of the N-methyl-d-aspartate receptor (NMDAR) (Nanney et al. 1986; Morhenn et al. 2004), the activities of EGFR and NMDAR in relation to wound healing in PD is unclear. However, there is evidence that EGF is a biomarker for cognitive decline in PD (Lim et al. 2016; Jiang et al. 2015; Chen-Plotkin et al. 2011; Turner et al. 2014). There have also been links between EGF and NMDA, specifically EGFR stimulation affecting NMDAR signaling (Abe and Saito 1992; Suina et al. 2018). 

### Hypothesis

We theorize that wound healing may be impaired in the context of PD based on NMDA and EGF activity in the central nervous system, and explore the current evidence regarding these signaling pathways in keratinocytes. Comparisons will be drawn with psoriasis, which demonstrates an increased rate of keratinization and wound healing. By contrast, if the skin of PD affected patients demonstrates decreased levels of EGFR and changes in various NMDAR subunit expression, the rate of reepithelization, and thus wound healing may be impaired.

### Evidence

#### The role of EGFR in keratinocytes

EGF is a polypeptide that stimulates cell growth, differentiation, and apoptosis across mammalian tissue types by binding to EGFR. EGFR is a tyrosine kinase transmembrane protein located on epidermal keratinocytes preferentially localized in the basal layer (Alavi et al. 2019). EGF is secreted in a paracrine manner by macrophages, fibroblasts, and platelets to stimulate keratinocyte proliferation and migration in early wound healing (Gibbs et al. 2000; Schneider and Wolf 2009). EGF availability and EGFR activity and localization have been extensively studied in the pathogenesis of impaired wound healing and in chronic wounds (Liu et al. 2012; Gorouhi et al. 2014; Raghunathan et al. 2021). Several studies cite that the use of EGFR inhibitors at wound sites cause decreased keratinocyte migration, re-epithelization, and rates of wound healing (Stoll et al. 1997; Tokumaru et al. 2000; Repertinger et al. 2004; Nakamura et al. 2001). Furthermore, reduced EGFR phosphorylation leads to reduced healing of induced wounds in the corneal epithelium of diabetic rats (Xu and Yu 2011). EGF secretion may be impaired or EGFR is not being sufficiently stimulated if wound healing is slower in patients with PD.

#### EGFR in psoriasis, a condition characterized by increased keratinocyte proliferation

Psoriasis is a chronic immune-mediated inflammatory disease characterized by abnormal cell cycling which results in hyperproliferation of keratinocytes. In contrast to conditions of impaired wound healing, EGF and EGFRs are overexpressed in psoriatic keratinocytes and thus play a role in the abnormal proliferation and differentiation in this process (Nanney et al. 1986). There is an increase in EGFRs in the upper layer of the epidermis of active psoriasis in comparison to the skin of individuals with normal skin, demonstrated by several studies (Nanney et al. 1986; Gottlieb et al. 1988; Elder et al. 1989). This overexpression has been linked to an observed increased rate of wound healing in psoriatic patients relative to age-matched controls (Morhenn et al. 2013a, b). The use of EGFR specific tyrosine kinase inhibitors such as ones used in cancer immunotherapies, i.e. erlotinib, have been associated with improvements in plaque-type psoriasis (Giroux Leprieur et al. 2010; Overbeck and Griesinger 2012; Oyama et al. 2012; Wierzbicka et al. 2006). In contrast to the high levels of EGFR in the epidermis of psoriatic patients, it was found that soluble serum EGFR levels were decreased and soluble serum EGF was increased in patients with psoriasis as compared to controls. The high levels of serum EGF and low levels of serum EGFR correlated with disease activity. It is theorized that the binding of EGF to EGFR in serum causes the decrease in serum EGFR, which in turn drives additional production of EGF (Flisiak et al. 2014). 

#### EGF in neurons as a biomarker for the progression of PD

In neurons, EGF acts a neurotrophic factor that appears to be a biomarker for the progression of PD. EGF in plasma may also be associated with PD progression demonstrated by Lim et al. showing that a low baseline plasma EGF was associated with lower cognition (Lim et al. 2016). Early PD patients were found to have low plasma EGF levels as compared to controls (Lim et al. 2016). The same study determined that advanced PD patients had elevated plasma EGF levels as compared to controls; however, other studies did not similarly suggest this increase in EGF with PD progression (Jiang et al. 2015). A study by Turner et al. also attested that higher serum EGF is associated with better executive functioning in PD patients and suggests that EGF may play a neuroprotective role in cognition in the context of PD (Turner et al. 2014). Additional studies contribute to the correlation of low plasma EGF levels with low baseline cognitive performance and the risk of cognitive decline in PD patients (Chen-Plotkin et al. 2011). Furthermore, lower levels of EGF were found in the substantia nigra lesions of rat brains. It was also demonstrated that lower levels of EGF and EGFR are found in postmortem brains of patients with PD as compared to controls (Iwakura et al. 2005). While serum EGF levels may not directly reflect epidermal EGF, it is possible that circulating EGF could modulate epidermal keratinocyte response, indicating a possible systemic influence. Alternatively, higher levels of epidermal EGF might contribute to increased serum EGF, but this exact relationship needs to be further investigated.

#### Altered EGFR in the keratinocytes of patients with PD

**Fig. 1:**
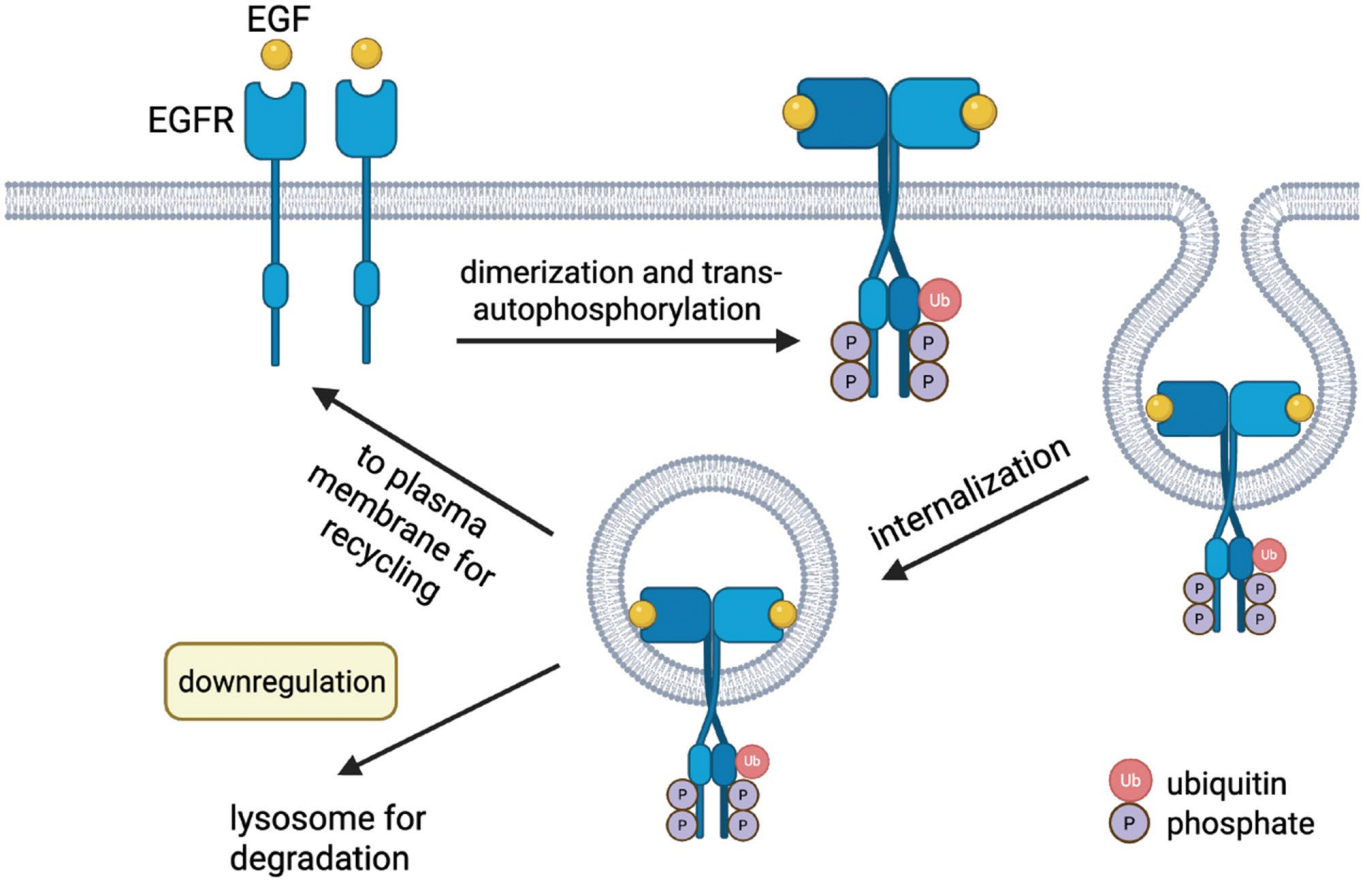
EGF may be downregulated in keratinocytes of patients with PD leading to impaired wound healing

Since the interactions of EGF and EGFR are downregulated in the brain and plasma of PD patients, this pathway may also be downregulated in epidermal keratinocytes contributing to impaired wound healing. Given the high levels of epidermal EGFR in psoriatic patients, it can be hypothesized that the keratinocytes in the epidermis of PD subjects have lower levels of EGFR expression than do the keratinocytes in the skin of normal subjects (Fig. 1). While there are no previous studies in current literature confirming the relationship between EGFR expression in PD patients’ keratinocytes, it is well-established that altered EGFR signaling plays a role in various dermatological conditions, including psoriasis. Given the potential for dysregulated EGFR signaling in PD, the hypothesis that EGFR levels may be altered in PD patients’ keratinocytes, thus impairing wound healing, warrants further exploration.

#### The role of NMDA

The integument and the central nervous system are both of ectodermal origin, and there is evidence that NMDARs are expressed in both neurons and keratinocytes (Morhenn et al. 1994; Denda et al. 2007). These receptors are a class of G protein-coupled ionotropic glutamate receptors that have important excitatory functions in the motor circuits of the basal ganglia (Hallett and Standaert 2004). Additionally, NMDARs of neurons play a role in long-term adaptive and regulatory signaling as well as in neural injury as a result of disease (Hallett and Standaert 2004). These receptors have been largely studied for the development of NMDAR antagonists to treat symptoms of PD. The role of NMDARs in keratinocytes have been less studied than that of neurons; however, there is evidence that NMDARs can influence epithelialization, and thus wound healing in keratinocytes (Nahm et al. 2004). 

#### NMDAR subunits

Structurally, seven NMDAR subunits have been identified, to contribute specific qualities to either tetrameric or pentameric receptor complexes. The nomenclature for these subunits have evolved, with GluN2, NR2, and N2 referring to the same subunit, for example. In this paper, we will use the currently accepted naming convention, “GluN2.” NMDAR complexes are composed of one or two GluN1 subunits, at least one GluN2 subunit, and less commonly, a GluN3 subunit (Chaffey and Chazot 2008). Additionally, there are four types of GluN2 subunits including GluN2A, GluN2B, GluN2C, and GluN2D (Glasgow et al. 2015). The complex of subunits form a channel that is activated by the binding of glutamate. The channel is highly permeable to Ca^2+^ and is blocked in a voltage dependent manner by Mg^2+^. The influx of Ca^2+^ upon the binding of glutamate initiates various intracellular second messenger cascades. The kinetics are dependent on the characteristics of the specific subunits, subunit combinations, as well as the location and quantity of NMDAR activations by glutamate (Nahm et al. 2004). For example, there are differences between NMDAR subunit compositions in the brain versus in keratinocytes. The brain’s NMDARs are composed of two glycine-binding (GluN1) subunits and two glutamate-binding (GluN2) subunits, while keratinocytes classically express GluN2B (Glasgow et al. 2015). In the CNS, Ca^2+^ entry through NMDARs lead to events such as gene expression, neural circuit development, synaptic plasticity, and cell signaling (Chaffey and Chazot 2008). 

#### NMDAR in PD

In PD, there are dopamine-glutamate imbalances within the striato-thalamo-cortical loop, reducing motor cortex stimulation. The reduction in dopamine causes of lack of inhibition of glutamatergic activity. This overstimulation at glutamatergic sites is thought to upregulate NMDAR activity in the brain of PD patients (Chaffey and Chazot 2008; Guo et al. 2017). Several studies demonstrate changes in expression levels of NMDAR subunits attributed to PD related dopamine depletion as well as L-DOPA usage (Zhang et al. 2019). The GluN2A to GluN2B subunit ratio was found to be increased in the post-mortem brain tissue of PD patients as well as in the striatum of rats and monkeys treated with L-DOPA (Mellone et al. 2015). A study by Gan et al. found GluN1 and GluN2B to be upregulated at the surface of the striatum (Gan et al. 2014). Another study demonstrated an increase in the GluN2D subunit in the striatum of L-DOPA treated rats (Mellone et al. 2019). For this reason, NMDA antagonists are widely studied and developed to treat both motor and non-motor symptoms of PD (Hallett and Standaert 2004). While the specific subunits that were upregulated were inconsistent across studies, a hallmark is that there are altered ratios of NMDAR subunits in PD. It is worthwhile to consider staining various subunits of NMDA to localize them as it relates to our hypothesis.

#### NMDARs in keratinocytes

NMDARs have also been identified by Morhenn and colleagues in non-neuronal tissues such as keratinocytes, and play a functional role in regulating Ca^2+^ entry into those cells, which in turn regulates keratinocyte growth and differentiation (Nahm et al. 2004; Morhenn et al. 1994, 2013a, b; Bikle et al. 2012). Glutamate was found in high concentrations in inflamed or wounded skin, indicating that the NMDARs in keratinocytes can be activated endogenously (Nahm et al. 2004; Fischer et al. 2009). Immunocytochemistry has been used to confirm the expression of the GluN1 subunit of the NMDAR in normal human keratinocytes cultured in vitro, in bioengineered skin constructs, as well as in keratinocyte epithelium of normal human skin and psoriatic skin (Nahm et al. 2004; Morhenn et al. 2004). GluN1 was chosen as it is a fundamental subunit of NMDARs; however, GluN2 subunits are also thought to be expressed in keratinocytes. Cell-to-cell contact signaling triggers surface expression of NMDARs as demonstrated by high levels of cell surface GluN1 expression in cells grown confluently compared to cells grown in isolation. While confluent keratinocytes exhibited perinuclear GluN1 distribution, isolated or non-confluent cells did not have cell surface GluN1 expression (Nahm et al. 2004). Immunocytochemistry confirmed that the GluN1 subunit was expressed in a polarized pattern in suprabasal keratinocytes of wounded, bioengineered skin constructs. After wounding, GluN1 was distributed in a clustered manner at the epidermal surface opposite to the direction in which keratinocytes were migrating. These results suggest that wounding alters NMDAR activation in keratinocytes involved in re-epithelialization, and that Ca^2+^ entry through NMDAR may influence keratinocyte proliferation and migration. The number and distribution of NMDARs can be a way of regulating Ca^2+^ entry and keratinocyte proliferation. In non-disease states, the process of re-epithelialization is suggested to be inhibited by NMDAR activation (Nahm et al. 2004). 

There is also evidence that squamous cell carcinoma lacks NMDAR expression as there was no GluN1 expression in malignant keratinocytes through staining (Nahm et al. 2004). However, the healthy, non-malignant keratinocytes surrounding squamous cell carcinoma demonstrated strong expression of GluN1, once again suggesting the role that NMDARs may play in keratinocyte differentiation and in the inhibition of growth that is absent in neoplastic proliferation (Nahm et al. 2004). 

#### NMDAR in psoriasis and evidence of increased rates of wound healing

Psoriatic skin demonstrates epidermal hyperproliferation and abnormal growth and differentiation of keratinocytes (Morhenn et al. 2013a, b). In a previous prospective study, biopsies were performed on both involved and uninvolved skin of subjects with psoriasis and compared with biopsies of the skin of normal controls. It was determined that both the involved and uninvolved skin of patients with psoriasis demonstrated faster wound healing compared to the skin of controls and that the NMDAR is abnormally expressed (Morhenn et al. 2013a, b). Based on immunohistochemistry, the GluN2C subunit of the NMDAR is downregulated in the basal cell layer of the skin of psoriatic patients as compared to controls. In contrast, the GluN2D subunit is more highly expressed in the basal cell layer of uninvolved epidermis of psoriatic patients, despite the noted increase in wound healing. These differences in the rates of subunit expression between involved and uninvolved epidermis of psoriatic patients and those of control patients may explain the observed lack of growth inhibition in psoriatic keratinocytes (Morhenn et al. 2013a, b). 

The downregulation of GluN2C subunits in the skin of psoriatic patients and the resulting increased wound healing may be partially explained by a separate study proposing that GluN2C subunit expression is necessary for the inhibition of growth after wound re-epithelialization and keratinocyte confluence. Tumor necrosis factor alpha (TNFα), shown to be a mediator of EGFR, upregulates the GluN2C mRNA in differentiated, normal keratinocytes to inhibit excessive growth (Morhenn et al. 2013a, b; Yoo et al. 2012; Wei et al. 2021). Psoriatic keratinocytes are resistant to TNFα induction of mRNA for the GluN2C subunit. TNFα was not able to inhibit the growth of psoriatic keratinocytes, resulting in their hyperproliferation (Morhenn et al. 2013a, b). 

#### Altered NMDARs affecting wound healing in PD

**Fig. 2:**
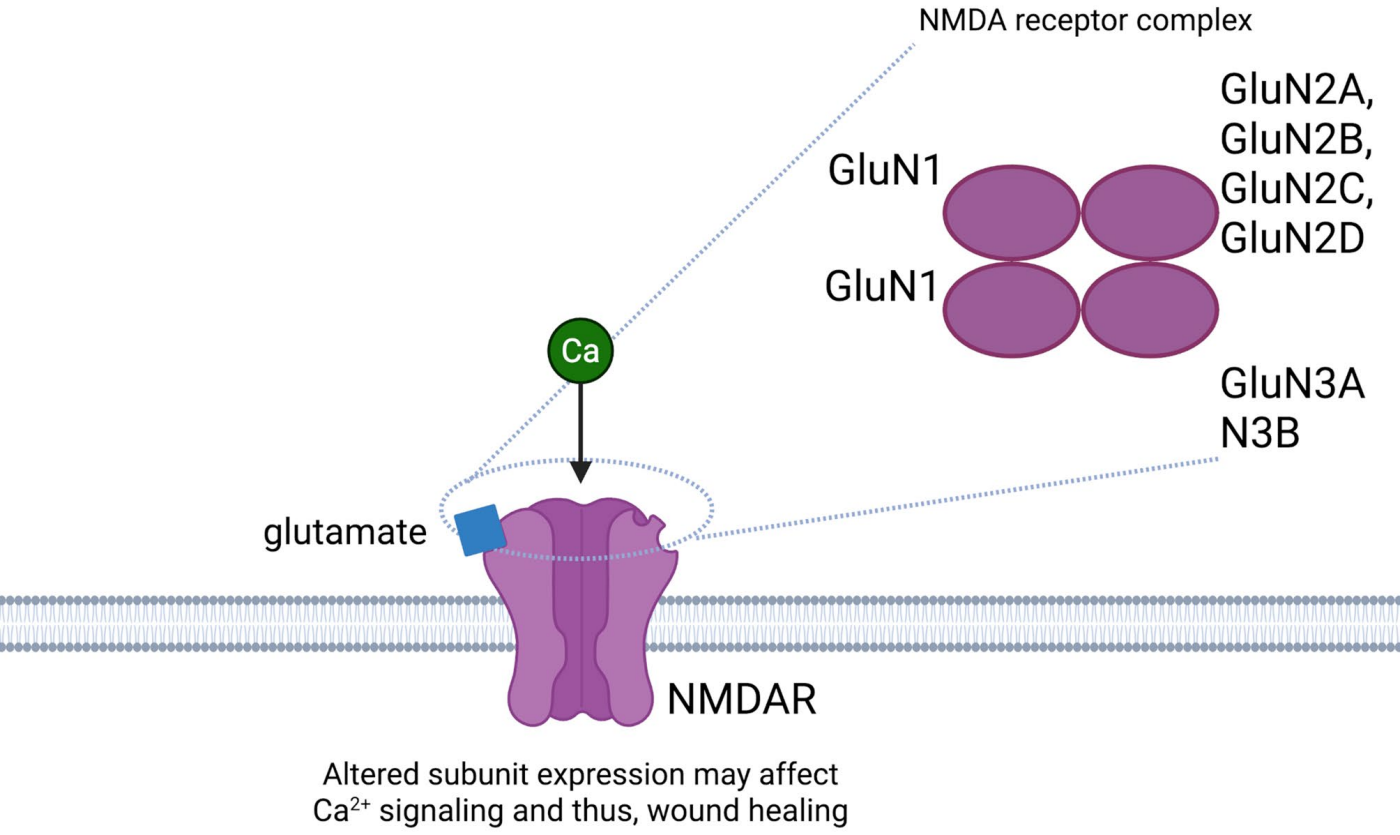
Altered NMDA Receptor Subunit Expression May Impair Wound Healing in PD. NMDAR complexes, which mediate calcium (Ca²⁺) entry into cells, are composed of one or two GluN1 subunits, at least one GluN2 subunit, and occasionally a GluN3 subunit. Activated by glutamate, found elevated in inflamed or wounded skin, these receptors are expressed in keratinocytes. They regulate proliferation and migration, which are processes essential for wound healing. The number and localization of NMDARs, as well as ratio of receptor subunits, influence Ca²⁺ signaling. In PD, altered subunit expression may disrupt this signaling and impair wound repair

The pathophysiology behind wound healing as it relates to PD has not been previously investigated. However, given the increase in neuronal NMDAR activation in PD leading to some hallmark Parkinsonian symptoms as well as the polarized expression of various NMDAR subunits in keratinocytes as it relates to psoriasis, it is reasonable to propose that there may be alterations in the number, localization, and ratios of the various NMDAR subunits in the keratinocytes of PD that could lead to impaired wound healing (Fig. 2).

#### Link between EGF and NMDA

**Fig. 3:**
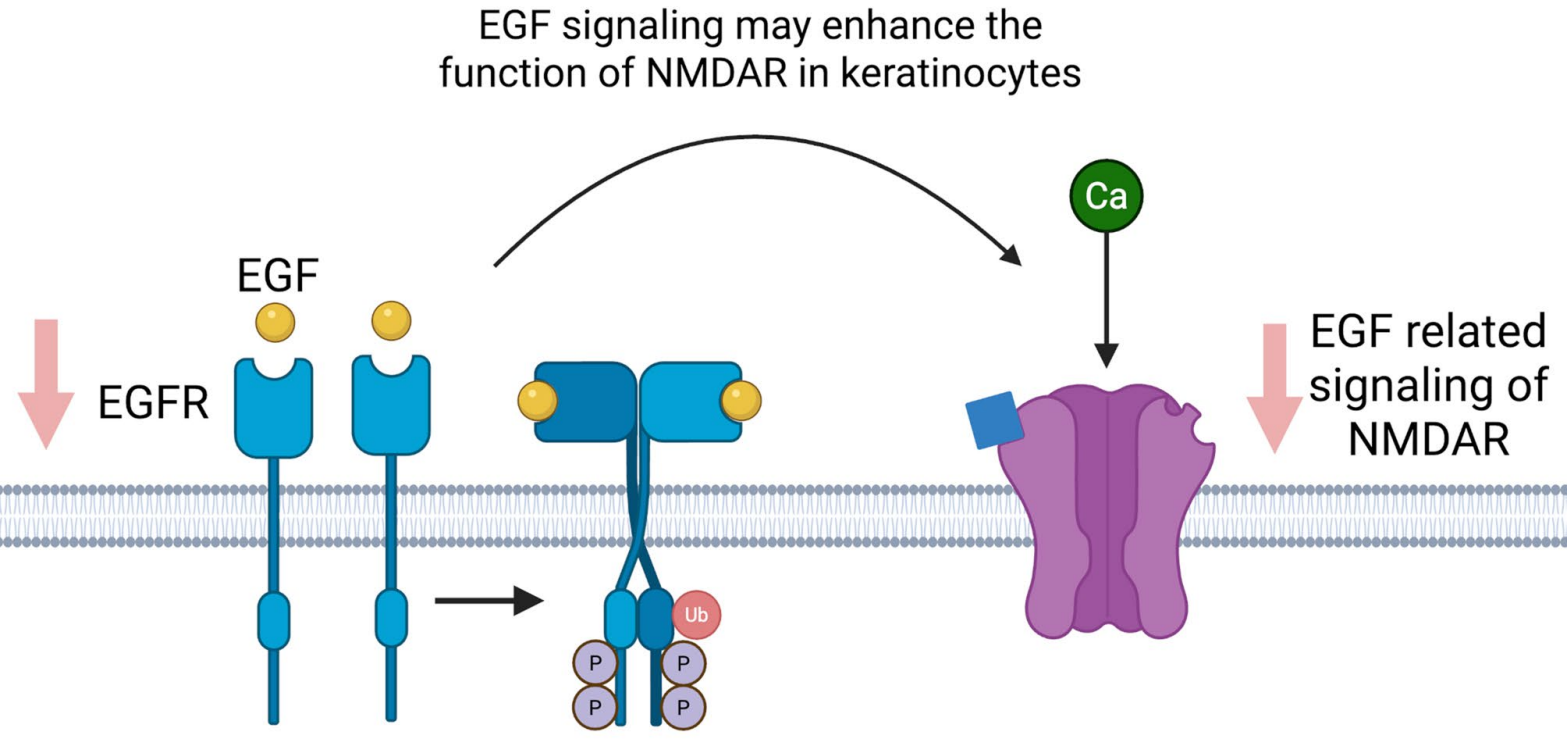
EGF-EGFR Signaling Enhances NMDAR Function in Keratinocytes. Simplified model of the potential link between EGF and NMDAR signaling in keratinocytes. EGF binds to and activates EGFR, which may enhance the function of NMDAR, leading to Ca²⁺ influx. In PD patients, decreased expression of EGFR in keratinocytes may lead to reduced EGF-mediated NMDAR activation, although the exact subunits involved and the downstream effects on wound healing remain unclear

Emerging literature suggests a functional link between EGF and NMDARs, potentially integrating these two signaling systems. In rat hippocampal neurons, EGF selectively enhances NMDAR-mediated increases in calcium, although the mechanism remains poorly understood (Abe and Saito 1992). Similarly, EGF stimulated EGFR in glioma cells which subsequently phosphorylated the carboxy terminal of the GluN2B subunit of NMDAR and enhanced glutamate-NMDAR signaling and then glioma cell migration (Suina et al. 2018). Another study also suggested that EGFR signaling upregulated NMDARs through phosphorylation of the GluN2B subunit (Tang et al. 2015). In these cases, EGF signaling enhances the function of NMDAR in neuronal tissue. This supports the notion that EGF signaling may also influence the regulation of NMDAR subunits in epidermal keratinocytes. Since we are hypothesizing that there are fewer EGFRs in keratinocytes of PD patients, there may be lower EGF-related stimulation of NMDAR, although which specific subunits are affected, how that affects the ratios between subunits, and subsequently affect the signaling for wound healing in PD is at this time unclear (Fig. 3).

## Implications and conclusions

We hypothesize that PD impairs skin wound healing through dysregulation of key molecular pathways. To test our hypothesis, we propose a clinical trial comparing wound healing in PD patients and age- and co-morbidity-matched controls. Excision wounds would be created, with longitudinal monitoring of closure rates and complications such as delayed healing. To minimize interference with healing, tissue samples would be collected only once at wound creation for RNA isolation and quantitative PCR (qPCR) analysis of NMDAR and EGFR expression. Immunohistochemistry (IHC) would validate protein expression and localization. By correlating receptor activity with healing outcomes and clinical variables such as PD duration and medication use, this study would clarify whether NMDAR and EGFR dysregulation contributes to impaired wound healing in PD and assess the influence of neurodegeneration on peripheral tissue repair. Controlling for co-morbidities, medications, pre-existing skin conditions, and other factors that may impair wound healing helps address potential confounders noted in the introduction. Integrating clinical data, such as PD duration and medication history, with molecular findings could clarify whether dysregulation varies across disease stages or treatments, ultimately strengthening evidence for NMDAR and EGFR signaling in impaired wound healing in PD.

Additionally, we completed a search within Gene Expression Omnibus (CEO) databases to obtain associative data regarding wound healing phenotypes and PD diagnoses. There are multiple GEO datasets (like GSE7621 and GSE20141) to study gene expression in substantia nigra tissue from PD patients and controls, identifying differentially expressed genes (DEGs) through methods like robust rank aggregation (RRA). Notably, analysis of GSE7621 revealed that wound healing processes were potentially enriched among DEGs in PD samples, suggesting shared molecular mechanisms between wound repair and PD pathophysiology. This information appears contrary to our hypothesis; however, existing gene repositories do not examine wound repair related genes specifically in keratinocytes. Additionally, there is no direct evidence from these GEO datasets showing differences in EGF, EGFR, NMDA, or NMDAR expression in the skin of PD patients compared to skin of controls, indicating that further work is indicated. If the hypothesis is true and patients with PD do demonstrate slower rates of wound healing in clinical trial, additional attention should be given to PD patients with dermatological concerns to ensure noninfecting and proper healing of their wounds.

## Data Availability

No datasets were generated or analysed during the current study.

## Abbreviations

PD: Parkinson’s Disease
EGF: Epidermal growth factor
NMDA: N-methyl-D-aspartate
EGFR: Epidermal growth factor receptor
NMDAR: N-methyl-d-aspartate receptor
TGF-alpha: Transforming growth factor alpha
